# Fast Estimation and Valid Statistical Inference for Mixed‐Effect Location‐Scale Models Using Variational Inference

**DOI:** 10.1002/sim.70640

**Published:** 2026-06-17

**Authors:** Brian Ping‐Huan Wu, Donald Hedeker

**Affiliations:** ^1^ Department of Statistics University of Chicago Chicago Illinois USA; ^2^ Department of Public Health Sciences University of Chicago Chicago Illinois USA

**Keywords:** longitudinal data, mixed effect models, robust standard errors, variational inference

## Abstract

With the development of modern technology, richer and more intensive longitudinal data are available for researchers to study more complex model structures. The Mixed‐Effect Location‐Scale Model (MELS) is useful for modeling the variance components in such data. However, fitting these models is computationally demanding using standard Maximum Likelihood estimation (MLE) or Markov Chain Monte Carlo (MCMC) methods. This paper introduces a fast, deterministic Variational Message Passing (VMP) algorithm for fitting MELS models. We derive the updates for the non‐conjugate components using a simplified Laplace approximation and employ a robust M‐estimation framework to construct sandwich estimators for valid frequentist inference. Simulation studies confirm our estimator is accurate and consistent. A real data example shows that our algorithm achieves point and interval estimates comparable to MLE in seconds, outperforming MLE (minutes) and MCMC (hours). This MELS‐VMP algorithm provides a computationally efficient and reliable alternative for model selection and inference on intensive longitudinal datasets.

## 1 Introduction

The advance of modern technology, such as smartphones and wearable devices, has led to the collection of intensive longitudinal datasets, i.e., longitudinal datasets with many observations per subject [1]. For example, Ecological Momentary Assessment (EMA) methods in psychology are used to study complex human behavior and processes [2]. These datasets are often collected through subjects responding to questions via smartphones or handheld computers. For example, in one such EMA study [3], participants were asked to report their mood when prompted many times a day during a week‐long measurement period. Alternatively, in physical activity studies [4], movement and exercise data are often collected regularly from wearable devices on subjects, providing researchers with hundreds or even thousands of observations per subject.

Intensive longitudinal datasets are rich enough to allow researchers to study the variance structure of the response, beyond the mean structure. For example, researchers in longitudinal psychology studies might want to study what covariates can lead to a more stable mood, i.e., lower variance. Traditional longitudinal modeling methodologies, such as linear mixed effect models, with the assumption of homogeneous variances both within and between subjects, are unable to address such questions. Mixed‐Effect Location‐Scale (MELS) model [5] offers an approach to such questions by modeling the variance with a log‐linear sub‐model including covariates that are of interest. Some examples include the mood reported by participants in a psychological study using the MELS model [3], investigating student performance by also considering the effect of schools [6] and considering heterogeneous random effect variances for general usage in meta‐analysis [7].

However, the intense computation required for fitting MELS models presents a significant obstacle. Current methods, including marginalizing the mixed effects by numerical approximations or exploring the likelihood function by Markov Chain Monte Carlo algorithms, both require a great deal of computation. These computational obstacles make model selection or refitting methods such as bootstrap impractical when dealing with real‐world datasets.

Variational inference, an idea that emerged from machine learning [8], has recently been used by statisticians in more general probabilistic modeling settings [9, 10]. Due to its flexibility and fast computational time, there has been significant interest in fitting latent variable models using VI. Simultaneously, theoretical properties of VI estimators have also been developed [11, 12, 13] to justify the use and allow for statistical inference. General algorithms for VI, such as Coordinate Ascent Variational Inference (CAVI) [8, 9], which can be implemented by Variational Message Passing (VMP) algorithms [10, 14], are widely used as they provide simple update steps within the algorithm. More general algorithms, such as Stochastic Variational Inference (SVI) [15] or Automatic Differential Variational Inference (ADVI) [16] have also been proposed to tackle more complicated modeling tasks. However, while black‐box algorithms like SVI and ADVI allow one to fit more complex and intractable models, they require well‐designed automatic differential systems. These algorithms may also introduce further approximations, potentially resulting in lower estimation precision as compared to VMP.

In this paper, we will introduce a VMP algorithm for fitting MELS models. Simulation results and an example on a real EMA dataset show that it can be much faster than traditional fitting methods (200× or even more on large datasets) while still providing empirically consistent estimators. Also, the flexibility of the VI framework can allow for more advanced MELS modeling, leading one to study more complex multi‐level model structures when there is scientific interest in doing so. Moreover, our algorithm can offer excellent starting values for MLE or MCMC algorithms for faster and more stable convergence.

This paper is structured as follows: Section 2 introduces the MELS model and current techniques for fitting it. Section 3 introduces the Variational Inference concept and the general algorithm and inference methods. In Section 4, we will illustrate our VMP algorithm for fitting MELS models, including an initialization process for the algorithm. In Section 5, we will show results from some simulation studies of the empirical properties of the algorithm, and a real data example to illustrate the performance of the algorithm as compared to existing methods. Finally, we will conclude with a discussion in Section 6.

## 2 Mixed Effect Location Scale Models

Consider the following simple Linear Mixed‐effects Model (LMM) for a longitudinal data y, which consists of N subjects with $n_{i}$ observations for each subject (i=1,…,N):

(1a) $$\begin{array}{ll} & y_{ij}=x_{ij}^{⊤}\beta +\nu_{i}+\varepsilon_{ij} \end{array}$$

(1b) $$\begin{array}{ll} & \nu_{i}∼𝒩(0,\sigma_{\nu }^{2}) \end{array}$$

(1c) $$\begin{array}{ll} & \varepsilon_{ij}∼𝒩(0,\sigma^{2}) \end{array}$$

Here, $y_{ij}$ is the $j^{th}$ ($j=1,\ldots ,n_{i}$) observation of subject i, i=1,…,N. The vector $x_{ij}\in {\mathbb{R}}^{p_{1}}$ is the covariate vector corresponding to the observation $y_{ij}$ and includes the intercept. $\beta \in {\mathbb{R}}^{p_{1}}$ is the vector of regression coefficients. The subject‐level random‐effects $\nu_{i}$ follow a $𝒩(0,\sigma_{\nu }^{2})$ distribution, while the residual errors $\varepsilon_{ij}$ follow a $𝒩(0,\sigma^{2})$ distribution. The variance of the random effects, $\sigma_{\nu }^{2}$, is the between‐subjects (BS) variance and describes the degree to which subjects differ from the overall average. The residual variance, $\sigma^{2}$, is the within‐subjects (WS) variance and captures the variability of observations within a subject.

In simple mixed‐effect models, we assume $\sigma_{\nu }^{2}$ and $\sigma^{2}$ to be constants. The MELS model relaxes these constraints by describing them in terms of log‐linear models:

(2a) $$\begin{array}{ll} & y_{ij}=x_{ij}^{⊤}\beta +\nu_{i}+\varepsilon_{ij} \end{array}$$

(2b) $$\begin{array}{ll} & \nu_{i}∼𝒩(0,\exp(U_{i}^{⊤}\alpha )) \end{array}$$

(2c) $$\begin{array}{ll} & \varepsilon_{ij}∼𝒩(0,\exp(W_{ij}^{⊤}\tau +\omega_{i})) \end{array}$$

(2d) $$\begin{array}{ll} & \omega_{i}∼𝒩(0,\sigma_{\omega }^{2}) \end{array}$$

The variance components for both the random effects and the residuals are heterogeneous now and can be modeled using fixed covariates, $U_{i}\in {\mathbb{R}}^{p_{2}}$ and $W_{ij}\in {\mathbb{R}}^{p_{3}}$ that both include an intercept. Note that $U_{i}$ can only include non‐time‐varying covariates. $\alpha \in {\mathbb{R}}^{p_{2}}$ is the corresponding regression vector for $U_{i}$ while $\tau \in {\mathbb{R}}^{p_{3}}$ is that for $W_{ij}$. Further, the random effect of the variance component, with fixed variance $\sigma_{\omega }^{2}$, is captured by the term $\omega_{i}$ to account for different variance intercepts for subjects. The exponential transformation term in the variance component models ensures that the variances remain positive. Here, $\nu_{i}$ is referred to as the random location effect and $\omega_{i}$ is referred to as the random scale effect, giving the MELS model its name. We will assume that these two random effects are independent of each other. This paper will stick to the definitions given in Equation (2), and the parameter space of interest is denoted by $\Theta =(\beta ,\alpha ,\tau ,\sigma_{\omega }^{2})$. In this paper, the example and model description are in terms of intensive longitudinal data; however, this model can also be applied to clustered data, with subjects nested within geographical locations, hospitals, schools, etc.

### 2.1 Frequentist Approach

Maximum likelihood estimation (MLE) is a common method for fitting MELS models in Equation (2). This approach is implemented in various programs such as MixRegLS [5], Merlin in Stata [17] and PROC NLMIXED in SAS [18]. In this approach, the random effects are first standardized as follows: $\theta_{1i}=\frac{\nu_{i}}{\sigma_{\nu }}$ and $\theta_{2i}=\frac{\omega_{i}}{\sigma_{\omega }}$. Here, $\theta_{1i}$ and $\theta_{2i}$ follow standard normal distributions. Then, we have:

$$y_{ij}|\theta_{1i}.\theta_{2i}=𝒩(x_{ij}^{⊤}\beta +\theta_{1i}\exp(U_{i}^{⊤}\alpha /2),\exp(W_{ij}^{⊤}\tau +\theta_{2i}\sigma_{\omega }))$$

We can then obtain the marginal likelihood of y by integrating out the standardized random effects $\Theta_{1}=(\theta_{11},\theta_{12},\ldots ,\theta_{1N})$ and $\Theta_{2}=(\theta_{21},\theta_{22},\ldots ,\theta_{2N})$.

(3) $$L(y)=\int \int f(y|\Theta_{1},\Theta_{2})\phi (\Theta_{1})\phi (\Theta_{2})d\Theta_{1}d\Theta_{2}$$

where ϕ(·) is the density function of the standard normal distribution.

However, there is no closed‐form solution for (3); the adaptive Gauss‐Hermite quadrature is commonly used to approximate the integral. This involves replacing the integral with a sum that is evaluated at a pre‐specified grid. In our example, if Q points are specified for each dimension, then the integral is approximated over a grid of size $Q^{2}$ for the two random effects. This can be very time‐consuming as the dataset grows, since an evaluation is required for every data point at each iteration.

In the fastregls [19] approach, they demonstrated that for a heteroskedastic model

$$\begin{array}{ll} & y_{i}=X_{i}^{⊤}\beta +\varepsilon_{i}\\ & \varepsilon_{i}∼𝒩(0,\exp(W_{ij}^{⊤}\tau +\omega_{i})) \end{array}$$

it can be shown that one can obtain the residuals $\hat{\varepsilon_{i}}$ from a least squares estimate $\hat{\beta }$. Asymptotically, these residuals follow the relationship $\log\hat{\varepsilon_{i}}=W_{i}^{⊤}\tau +\Psi_{i}$, where $\Psi_{i}∼\log\chi_{1}^{2}$. While this asymptotic property can be extended to achieve much faster computation for MELS‐type structures by eliminating the need for a two‐dimensional Gauss‐Hermite quadrature grid, it comes with a trade‐off in flexibility. However, it is important to note that this specific formulation, as implemented in the fastregls model, typically assumes a constant BS variance. While this approach is highly efficient for many applications, it does not include the random location effect sub‐model that allows the between‐subject variance to vary with covariates. Consequently, while this method offers a fast alternative for models with homoscedastic random effects, the MLE, Bayesian, and VMP approaches discussed in this paper provide a more flexible framework for researchers who require modeling of the BS variance structure.

### 2.2 Bayesian Approach

Bayesian modeling provides a flexible structure for fitting more complex MELS models. Some examples [20, 21] include 3‐level MELS models where level‐1 measurements are nested in level‐2 (e.g., observations nested within measurement waves) and level‐3 (e.g., measurement waves nested within subjects) units, are implemented in BUGS [22] and Stan [23]. Another extension is the multivariate normal MELS models [24] fitted by brms [25].

In these Bayesian modeling examples, all elements in Θ are treated as random and assigned prior distributions. A common tool for obtaining their posterior distributions for subsequent statistical inference is Markov Chain Monte Carlo (MCMC). MCMC begins with an initial random sample $\theta_{0}$, drawn from an initial distribution $q(\theta_{0}|x)$ where θ represents the latent variables of interest and x represents the data. A carefully chosen random transition operator $\theta_{t}∼q(\theta_{t}|\theta_{t-1},x)$ is then iteratively applied to subsequent random draws. After running this procedure many times, the final outcome $\theta_{T}$ will be a random variable that converges to the exact posterior p(θ|x). While MCMC offers a flexible structure for multi‐level modeling, it does not guarantee how many iterations are needed for convergence. In practice, obtaining a good approximation may require running the algorithm for a long time, which does not solve the issue of the computational time required for fitting MELS models.

## 3 Variational Inference

Another approach to fitting MELS models by Bayesian modeling is Variational Inference (VI). Unlike MCMC, which approximates the posterior by iterative sampling, VI introduces surrogate distributions q(θ) to approximate the posterior distribution p(θ|x) by minimizing the KL‐divergence between q(θ) and p(θ|x).

(4) $$\text{KL}(q(\theta )\|p(\theta |x))={𝔼}_{q}[\log q(\theta )]-{𝔼}_{q}[\log p(\theta |x)],$$

where ${𝔼}_{q}[\cdot ]$ denotes taking the expectation with respect to q. These surrogate distributions are chosen from a family of densities (e.g., Normal densities) that are parameterized by variational parameters which can be learned from the data.

Equation (4) can further be expressed as:

(5) $$\begin{array}{ll} \text{KL}(q(\theta )\|p(\theta |x)) & ={𝔼}_{q}[\log q(\theta )]-{𝔼}_{q}[\log p(\theta |x)] \end{array}$$

(6) $$\begin{array}{ll} & ={𝔼}_{q}[\log q(\theta )]-𝔼[\log p(\theta ,x)]+\log p(x), \end{array}$$

since logp(x) does not depend on q. This reveals the dependence of the KL‐divergence on logp(x), making it intractable in most cases. For example, in our MELS model Equation (3), it shows that the log likelihood relies on the computation of no closed‐form integrals. To address this, we define the evidence lower bound (ELBO) as:

(7) $$\begin{array}{l} \text{ELBO}(q)={𝔼}_{q}[\log p(\theta ,x)]-{𝔼}_{q}[\log q(\theta )] \end{array}$$

And by doing some algebra, we can see that

$$\begin{array}{ll} \text{ELBO}(q) & =\log p(x)-\log p(x)+{𝔼}_{q}[\log p(\theta ,x)]-{𝔼}_{q}[\log q(\theta )]\\ & =\log p(x)-\text{KL}(q(\theta )\|p(\theta |x)). \end{array}$$

Since the KL‐divergence is always non‐negative, we obtain a lower bound for the marginal log likelihood logp(x)≥ELBO(q). By maximizing the ELBO, the gap between the variational approximation and the true posterior is reduced.

In this paper, the focus will be on the Mean Field Variational Inference (MFVI) family [9]. That is, an assumption that all $q_{k}(\theta_{k})$ distributions are independent will be made, with k=1,…K representing all latent variables:

(8) $$p(\theta_{1},\theta_{2},\ldots ,\theta_{K}|y)\approx q_{1}(\theta_{1})q_{2}(\theta_{2})\cdots q_{K}(\theta_{K}).$$

By making this assumption, one can derive a Coordinate Ascent Variational Inference (CAVI) algorithm for optimizing the ELBO. It can be shown that [9] given a parameter set $\theta =(\theta_{1},\theta_{2},\ldots ,\theta_{K})$, the optimal density for any $\theta_{k},\;k=1,\ldots ,K$ is:

(9) $$\begin{array}{ll} q_{k}^{*}(\theta_{k}) & \propto \exp\{{𝔼}_{q-\theta_{k}}\log p(x,\theta )\} \end{array}$$

(10) $$\begin{array}{ll} & \propto \exp\{{𝔼}_{q-\theta_{k}}\log p(\theta_{k}|𝒮(\theta_{k})\}, \end{array}$$

where ${𝔼}_{q-\theta_{i}}$ denotes taking expectations over all $q_{i}(\theta_{i})$ except $q_{k}(\theta_{k})$, and $𝒮(\theta_{k})$ is the Markov Blanket of $\theta_{k}$. The Markov blanket of a variable is defined by the set that contains the parents, children, and the co‐parents of that variable in the Probabilistic Graphical Model (PGM). By starting from some initial θ values, we can iteratively update each $\theta_{k}$ until the ELBO meets some convergence criterion. It is guaranteed that a local optimum will be found when the algorithm converges. This is an EM‐like approach, although all parameters are considered as random variables, with expectations being taken for all variables to update their distribution parameters. Thus, similar to EM, it provides local optima.

When a conjugate or conditionally conjugate prior for $\theta_{k}$ is used, then $q_{k}^{*}(\theta_{k})$ will belong to a recognizable density family and have closed‐form update equations. Suppose that $p(\theta_{k})$ belongs to an exponential family and a complete or conditional conjugate prior is used, then one can write the distribution as:

$$p(\theta_{k}|\theta_{-k},y)=h(\theta_{k})\exp\{\eta_{k}{\left(\theta_{-k},y\right)}^{⊤}t(\theta_{k})-a(\eta_{k}(\theta_{-k},y))\},$$

where $t(\theta_{k})$ is the sufficient statistic, $\eta_{k}{\left(\theta_{-k},y\right)}^{⊤}$ is the natural parameter, $h(\theta_{k})$ is the base measure, and a(·) is the log‐normalizer. We can then substitute this representation into the general update rule in Equation (9).

$$\begin{array}{ll} {𝔼}_{q-\theta_{k}}[\log p(\theta_{k}|\theta_{-k},y)] & ={𝔼}_{q-\theta_{k}}[\log h(\theta_{k})+\eta_{k}{\left(\theta_{-k},y\right)}^{⊤}t(\theta_{k})\\ & \;-a(\eta_{k}(\theta_{-k},y))]\\ & =\log h(\theta_{k})+{𝔼}_{q-\theta_{k}}{\left[\eta_{k}(\theta_{-k},y)\right]}^{⊤}t(\theta_{k})\\ & \;-{𝔼}_{q-\theta_{k}}[a(\eta_{k}(\theta_{-k},y))]. \end{array}$$

Now, we can substitute this result back into the expression for $q_{k}^{*}(\theta_{k})$:

$$\begin{array}{ll} q_{k}^{*}(\theta_{k}) & \propto \exp\{\log h(\theta_{k})+{𝔼}_{q-\theta_{k}}{\left[\eta_{k}(\theta_{-k},y)\right]}^{⊤}t(\theta_{k})\\ & \;-{𝔼}_{q-\theta_{k}}[a(\eta_{k}(\theta_{-k},y))]\}\\ & \propto h(\theta_{k})\exp\{{𝔼}_{q-\theta_{k}}{\left[\eta_{k}(\theta_{-k},y)\right]}^{⊤}t(\theta_{k})\}. \end{array}$$

We can see that $q_{k}^{*}(\theta_{k})$ is in the same exponential family as the original $p(\theta_{k})$ with the same base measure and the sufficient statistic. Therefore, the new natural parameters $\eta_{k}^{*}$ can be easily identified:

(11) $$\eta_{k}^{*}={𝔼}_{q-\theta_{k}}[\eta_{k}(\theta_{-k},y)].$$

This gives the updates when the conjugacy exists.

### 3.1 Variational Parameter Updates for Non‐Conjugate Models

Unfortunately, the conjugacy does not always hold. For example, in the MELS model, since the variance component is modeled using a log‐linear model, the non‐linear exponential transformation breaks the conjugacy. Therefore, there are no closed‐form update functions for updating the values of α, τ, and the $\omega_{i}$'s.

We can assign Normal variational distributions to non‐conjugate variables and use Laplace or delta‐method approximations to derive the updates for the mean and variance parameters of the variational distributions [26]. We will focus on the Laplace approximation, in which we approximate the mean and variance of the true posterior by the gradient and Hessian of the ELBO value. The variational variance of the normal distribution is approximated by the curvature of the current ELBO [27], and the mean is then updated by a single Newton step from the mean of the previous iteration [28]. These results are derived through a sequence of matrix algebraic operations. To be more detailed, for $q_{k}(\theta_{k})=𝒩(\mu_{\theta_{k}}^{q},\sum_{\theta_{k}}^{q})$, the update function can be obtained by the following simplified Laplace approximation:

(12a) $$\begin{array}{ll} & L(\theta_{k})={𝔼}_{q}[\log p(\theta_{k}|𝒮(\theta_{k}))], \end{array}$$

(12b) $$\begin{array}{ll} & \sum_{\theta_{k}}^{q}\leftarrow {\left\{-H_{\mu_{\theta_{k}}^{q}}L(\theta_{k})\right\}}^{-1}, \end{array}$$

(12c) $$\begin{array}{ll} & \mu_{\theta_{k}}^{q}\leftarrow \mu_{\theta_{k}}^{q}+\sum_{\theta_{k}}^{q}{\left[D_{\mu_{\theta_{k}}^{q}}L(\theta_{k})\right]}^{⊤}, \end{array}$$

where $H_{\mu_{\theta_{k}}^{q}}$ and $D_{\mu_{\theta_{k}}^{q}}$ denote taking the Hessian and gradient with respect to $\mu_{\theta_{k}}^{q}$, respectively. Therefore, this approach is indeed seeking a Normal distribution to fit the quadratic approximation of the ELBO with respect to $\theta_{k}$. These results provide the basis for deriving all the updating equations required for the VMP algorithm to fit the MELS model.

### 3.2 Frequentist View of Variational Inference

While VI is rooted in Bayesian methodology for approximating intractable posterior distributions, it can also be connected with the Expectation‐Maximization (EM) algorithm [9], a classic frequentist tool for obtaining MLE solutions for latent variable models. The “E‐step”, which requires computing an expectation of the latent variables, is often intractable in EM algorithms. VI then offers a scalable alternative by replacing this intractable step with a tractable optimization step with a simpler family of surrogate posteriors by maximizing the ELBO. This establishes VI as a generalization of EM for approximating the MLE. From this perspective, the prior distributions in the models act as regularization terms akin to penalized likelihood methods.

This frequentist viewpoint allows asymptotic theory to be applied to VI estimators. A variational Bernstein‐von Mises theorem [12] has been established, which proves that under a general set of conditions, the point estimates derived from VI are consistent and asymptotically normal. A theoretical guarantee is also proved [29] for using non‐parametric bootstraps for constructing confidence intervals for VI estimators. Furthermore, one can connect VI to M‐estimators [13], providing a formal recipe for deriving the large sample properties for practical usage.

These developments are crucial for the uncertainty quantification of the VI estimators, since it is a well‐documented phenomenon that, due to the mean field assumption [9, 30], naively using the variational posterior tends to underestimate the true posterior variance and leads to unreliable inference results. The M‐estimation framework, e.g., considers the Variational approximation as a profile M‐estimator, then introduces the classic sandwich estimator under model‐misspecification to reintroduce the correlations for robust standard errors for the parameters of interest. Further, the non‐parametric bootstrap can also take advantage of the precise point estimates. By using these classic frequentist methodologies, we can construct reliable confidence intervals, allowing us to leverage the speed of VI without sacrificing statistical rigor. In this work, we explicitly adopt this frequentist perspective. We treat the mode of the variational distribution as the point estimation for the true parameter value and rely on these robust frequentist methodologies for all subsequent simulation and inference, ensuring our conclusions remain valid despite the approximations inherent in the mean field approach. A detailed explanation of how to apply these methods after we fit the MELS model by MFVI will be described in Section 4.3.

## 4 The Variational Message Passing Algorithm for MELS Models

**FIGURE 1:**
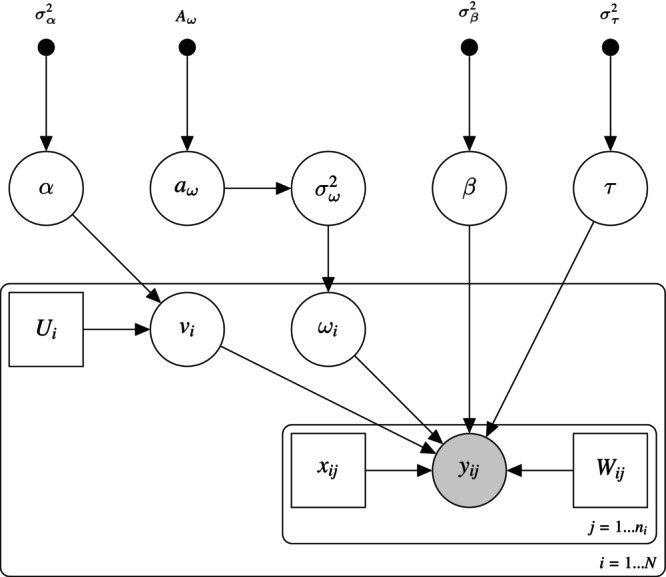
Probabilistic Graphical Model of the full Bayesian MELS model, representing the likelihood from Equation (2) and the priors from Section 4.1. Shaded circles are observed data ($y_{ij}$); clear circles are latent variables ($\beta ,v_{i},$ etc.); rectangles are fixed covariates ($x_{ij},$ etc.); and small bullets are fixed hyperparameters ($\sigma_{\beta }^{2},$ etc.).

For the MELS model defined in Equation (2), a common approach to represent this kind of latent variable model is to use a Probabilistic Graphical Model (PGM). The PGM for the full Bayesian MELS model is shown in Figure 1.

The PGM provides a blueprint for the proposed VMP algorithm, which is an implementation of CAVI. It works by iteratively optimizing the variational parameters $q_{k}(\theta_{k})$ for one variable at a time, while holding all others fixed. The visualization of PGM then provides a clear view of the Markov blanket of each variable, thus identifying the variables involved in updating the variational parameters of one variable. For example, when updating q(β), the parent is $\sigma_{\beta }^{2}$, the children are y, and the co‐parents are X,ν,τ,W, and ω. Therefore, the update must be a function of the expectations over these variables. The PGM thus serves as a map that identifies conditional dependencies that dictate which message, in the form of expectations, must be passed between variables to iteratively optimize the ELBO.

The VMP algorithm first sets the priors of Θ, the parameters of interest, as follows:

$$\begin{array}{ll} & \beta ∼𝒩(0,\sigma_{\beta }^{2}),\;\;\alpha ∼𝒩(0,\sigma_{\alpha }^{2}),\;\;\tau ∼𝒩(0,\sigma_{\tau }^{2}),\\ & \sigma_{\omega }^{2}∼\text{Half‐Cauchy}(A_{\omega }^{2}), \end{array}$$

where $\sigma_{\beta }^{2}$, $\sigma_{\alpha }^{2}$, $\sigma_{\tau }^{2}$ and $A_{\omega }$ are all set to a large number (e.g., $10^{4}$) to specify weakly‐informative priors in our examples. While the Half‐Cauchy prior is non‐conjugate, one can use a hierarchical structure of two Inverse‐Gamma priors to replace it [31]:

$$\begin{array}{cc} \sigma_{\omega }^{2} & ∼\text{Inverse‐Gamma}(1/2,1/a_{\omega }),\\ & a_{\omega }∼\text{Inverse‐Gamma}(1/2,1/A_{\omega }^{2}). \end{array}$$

The variational distributions of the parameters will be set as follows:

$$\begin{array}{ll} & q(\beta )=𝒩(\mu_{\beta }^{q},\sum_{\beta }^{q}),\;\;q(\alpha )=𝒩(\mu_{\alpha }^{q},\sum_{\alpha }^{q}),\;\;q(\tau )=𝒩(\mu_{\tau }^{q},\sum_{\tau }^{q})\\ & q(\nu_{i})=𝒩(\mu_{\nu_{i}}^{q},\sum_{\nu_{i}}^{q}),\;\;i=1,\ldots ,N\\ & q(\omega_{i})=𝒩(\mu_{\omega_{i}}^{q},\sum_{\omega_{i}}^{q}),\;\;i=1,\ldots ,N\\ & q(\sigma_{\omega }^{2})=\text{Inverse‐Gamma}(A_{\sigma_{\omega }^{2}}^{q},B_{\sigma_{\omega }^{2}}^{q}),\\ & \;q(a_{\omega })=\text{Inverse‐Gamma}(A_{a_{\omega }}^{q},B_{a_{\omega }}^{q}). \end{array}$$

### 4.1 Algorithm

The entire VMP algorithm is shown in Algorithm 1. For algebraic simplicity, define $h_{ij}$ as:

$$\begin{array}{cc} h_{ij} & ={𝔼}_{q}[{\left(y_{ij}-X_{ij}^{⊤}\beta -\nu_{i}\right)}^{2}]\\ & ={\left(y_{ij}-X_{ij}^{⊤}\mu_{\beta }^{q}-\mu_{\nu_{i}}^{q}\right)}^{2}+X_{ij}^{⊤}\sum_{\beta }^{q}X_{ij}+\sum_{\nu_{i}}^{q}. \end{array}$$

$\psi_{ij}^{\tau }$, $\psi_{i}^{\omega }$ and $\psi_{i}^{\alpha }$ are also defined as:

$$\begin{array}{ll} & \psi_{ij}^{\tau }={𝔼}_{q}[\exp(-W_{ij}^{⊤}\tau )]=\exp(-W_{ij}^{\tau }\mu_{\tau }^{q}+\frac{1}{2}W_{ij}^{⊤}\sum_{\tau }^{q}W_{ij}),\\ & \psi_{i}^{\omega }={𝔼}_{q}[\exp(-\omega_{i})]=\exp(-\mu_{\omega_{i}}^{q}+\frac{1}{2}\sum_{\omega_{i}}^{q}),\\ & \psi_{i}^{\alpha }={𝔼}_{q}[\exp(-U_{i}^{⊤}\alpha )]=\exp(-U_{i}^{⊤}\mu_{\alpha }^{q}+\frac{1}{2}U_{i}^{⊤}\sum_{\alpha }^{q}U_{i}). \end{array}$$

Note that these are the terms that appear in the variance components of Normal distributions.

The initialization of Algorithm 1 is described in the next subsection. For q(β), $q(\nu_{i})$'s, $q(\sigma_{\omega }^{2})$ and $q(a_{\omega })$, Equation (11) is used since they are conjugate in the model. Note that while the log‐linear variance structure makes the entire model non‐conjugate, the updates for q(β) and $q{\left(\nu_{i}\right)}^{\prime }s$ resolve to a closed form identical to a standard Normal‐Normal update. We therefore refer to them as conjugate updates to distinguish them from those requiring the Laplace approximation. And for q(α), q(τ), and $q(\omega_{i})$, the Laplace approximation in Equation (12) is used to update the variational parameters due to the non‐conjugacy. The full derivation can be found in the Appendix. The ELBO is calculated after every iteration, and the derivation can also be found in the Appendix. The program stops when it meets the following stopping criterion: If the difference of ELBO for the current iteration and the previous iteration is less than the absolute value of the current ELBO times a pre‐specified tolerance, which is $10^{-6}$ in our paper. We can check whether the algorithm has converged by tracking this criterion.

ALGORITHM 1 **Variational message passing for the MELS model.**

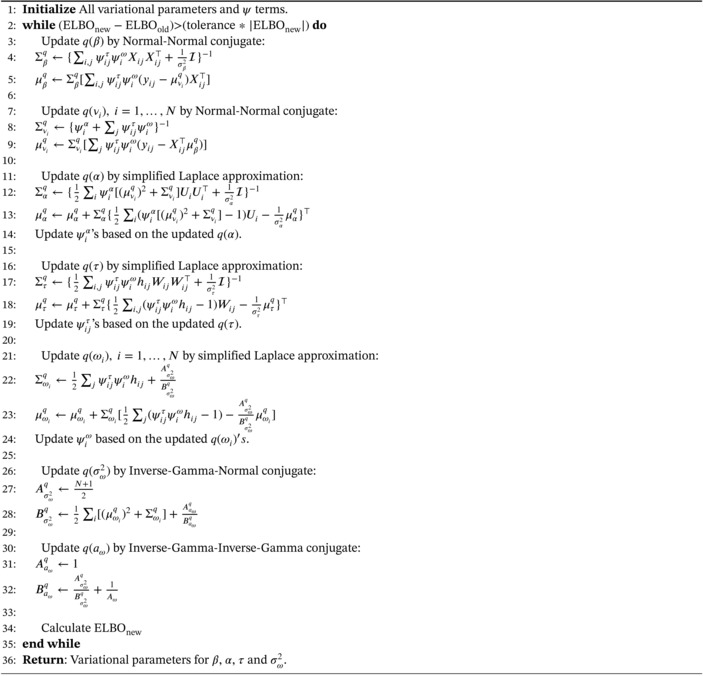

### 4.2 Initialization

For the initialization of the variational parameters, we will make use of the results from simple LMMs to get reasonable starting values for faster convergence in the algorithm and better numerical stability. Consider the model in Equation (1), we can use an existing program such as lme4 [32] to obtain initial estimates for the mean model parameters $\hat{\beta }$ and the random location effects $\hat{\nu_{i}}$'s. Second, we can use the residuals of this model $\hat{\varepsilon_{ij}}$ to initialize the within‐subject scale parameters. This is done by fitting a linear regression of $\log(\hat{\varepsilon_{ij}^{2}})$ on the covariates W; this provides initial values for the τ parameters. The random effects $\omega_{i}$'s are initialized by the mean and variance of the $n_{i}$ residuals, denoted as $\hat{\omega_{ij}}$, for each subject i. In a similar manner, the between‐subject scale parameters α are initialized by regressing $\log(\hat{\nu_{i}})$'s on the covariates U. Finally, the variances of $q(\nu_{i})$'s and $q(\omega_{i})$'s are set to some pre‐identified neutral values, e.g., values of 0.1. Further details are provided in Algorithm 2. This initialization ensures that all variational parameters begin in a reasonable part of the parameter space and enhances the numerical stability of the algorithm.

ALGORITHM 2 **Initialization of the VMP for the MELS Model.**

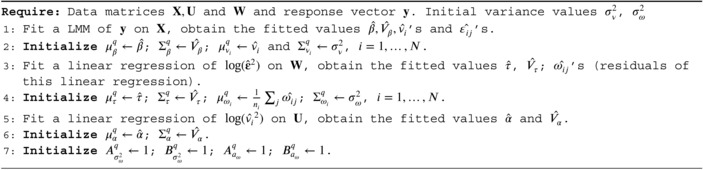

### 4.3 Statistical Inference

As noted in Section 3.2, the intervals derived directly from the VMP posterior approximations are known to underestimate the true parameter variances. To obtain reliable, frequentist‐style confidence intervals for our VMP estimates, fixed effects estimates as β,α, and τ, we adopt the non‐parametric bootstrap method [29] and the M‐estimation framework [13]. The idea of using a non‐parametric bootstrap for constructing valid confidence intervals around the mode of the variational distribution is simple: Although MFVI does not give reliable uncertainty quantification, it is good at approximating the true mode of the true posterior distribution, which can be thought of as the MAP estimate for the true parameter value under the frequentist perspective. Therefore, we can sample the subjects with replacement to preserve the correlation structure and fit the MELS model through our MELS‐VMP algorithm multiple times. Then we can collect the modes of all fitted variational distributions to construct the non‐parametric bootstrap confidence interval for our parameters of interest.

The profiled M‐estimator framework formally connects the estimators obtained by MFVI to a class of estimators known as profile M‐estimators, providing a theoretical basis for constructing robust, asymptotic standard errors. For any given set of global parameters, mean field VI algorithms search for the optimal local parameters, which creates a profile objective function. Our VMP estimator is an example that maximizes this profiled function. The idea is to obtain the per‐subject ELBO by factorizing each subject's contribution to the ELBO, then profile out their estimated random effects and the global variance parameters. Finally, adopt the M‐estimation framework to obtain the robust sandwich asymptotic covariance matrix of the fixed effects. It is important to note that because the local parameters are profiled out of this objective function, this specific sandwich formula is designed exclusively to adjust the asymptotic variance of the global parameters and does not yield adjusted standard errors for the latent variables.

To apply this to our MELS‐VMP algorithm, we divide our parameters into two sets: The global parameters $\theta =\{\mu_{\beta }^{q},\mu_{\alpha }^{q},\mu_{\tau }^{q}\}$ that are parameters of main interest, and the local parameters $\zeta_{i}=\{\mu_{\nu_{i}}^{q},\mu_{\omega_{i}}^{q}\}$ which are the parameters that we are profiling out. We then define the per‐subject ELBOs $L_{i}(\theta ,\zeta_{i}),\;i=1,\ldots ,N$ as the sum of all terms in the overall ELBO that depend on the data $y_{i}$ or the local parameters $\zeta_{i}$.

$$\begin{array}{cc} L_{i}(\theta ,\zeta_{i}) & ={𝔼}_{q}[\log p(y_{i}|\beta ,\nu_{i},\tau ,\omega_{i})]+{𝔼}_{q}[\log p(\nu_{i}|\alpha )]\\ & \;+{𝔼}_{q}[\log p(\omega_{i}|\sigma_{\omega }^{2})]. \end{array}$$

We ignore the entropy terms ${𝔼}_{q}[\log q(\nu_{i})]$ and ${𝔼}_{q}[\log q(\omega_{i})]$ since they do not depend on the global parameters. The full sandwich estimator can be constructed by summing all per‐subject components derived from $L_{i}$'s.

The meat of the sandwich, $\hat{B}$, captures the empirical variance of the gradients of $L_{i}$. It is computed as

$$\begin{array}{ll} & \hat{B}=\overset{N}{\underset{i=1}{\sum }}\hat{G_{i}}{\hat{G_{i}}}^{⊤},\\ & \hat{G_{i}}={\left.\frac{\partial }{\partial \theta }L_{i}(\theta ,\zeta_{i})\right|}_{\hat{\theta },\hat{\zeta }}. \end{array}$$

Note that $\hat{G_{i}}$ is the gradient of $L_{i}$ with respect to the global parameters θ evaluated at the final converged VMP estimates. The bread Â represents the total observed information of the profiled ELBO, which is an adjusted Hessian matrix:

$$\begin{array}{ll} & Â=\overset{N}{\underset{i=1}{\sum }}\hat{H_{i}}+\frac{\partial^{2}}{\partial \theta^{2}}L_{\text{priors}}(\hat{\theta }),\\ & \hat{H_{i}}={\left[\frac{\partial^{2}}{\partial \theta^{2}}L_{i}-\frac{\partial^{2}}{\partial \theta \partial \zeta }L_{i}{\left(\frac{\partial^{2}}{\partial \zeta^{2}}L_{i}\right)}^{-1}\frac{\partial^{2}}{\partial \zeta \partial \theta }L_{i}\right]}_{\hat{\theta },\hat{\zeta }}. \end{array}$$

The terms $\hat{H_{i}}$'s are correcting the naive Hessian under the mean field assumption, introducing back the relations between the global and local parameters. $L_{\text{priors}}(\hat{\theta })$ is the Hessian of the log‐priors for the global parameters, which is a simple diagonal matrix $\text{diag}(-1/\sigma_{\beta }^{2},-1/\sigma_{\alpha }^{2},-1/\sigma_{\tau }^{2})$, and have mild influence when the data have enough evidence. Finally, we can obtain the robust sandwich asymptotic covariance matrix for the fixed effects:

$$\hat{V}={Â}^{-1}\hat{B}{Â}^{-1}.$$

Let $P=p_{1}+p_{2}+p_{3}$ denote the number of global parameters in θ. The subject‐specific gradient $\hat{G_{i}}$ is then a P×1 vector. Consequently, the meat matrix $\hat{B}$, the adjusted Hessian bread matrix Â, and the final robust asymptotic covariance matrix $\hat{V}$ are all P×P symmetric matrices.

Many of these components, such as $\frac{\partial^{2}}{\partial {\mu_{\beta }^{q}}^{2}}L_{i}$, were derived in previous algorithm updates. By assembling these pieces, one can construct the final covariance matrix $\hat{V}$, and the square root of its diagonal elements provides the robust standard errors used in the Wald intervals, allowing one to make valid frequentist inference results. The full algebraic derivations of each gradient and Hessian component are provided in the Appendix.

By taking the square root of the diagonal elements of $\hat{V}$, we obtain the robust standard error for each global parameter. For any parameter $\theta_{i}$ in the global parameter set θ, we use the mode of the variational distribution, $\hat{\theta_{i}}$, as the point estimate of $\theta_{i}$. The robust standard error, $\sqrt{{\hat{V}}_{ii}}$, is the square root of the i‐th diagonal value of $\hat{V}$. The robust (1−α)% Wald confidence interval is then constructed as

$$\hat{\theta_{i}}\pm Z_{\alpha /2}\sqrt{{\hat{V}}_{ii}}$$

where $Z_{\alpha /2}$ is the (1−α/2) quantile of the standard normal distribution.

## 5 Results

In this section, we will present several simulations and an example using our VMP algorithm for MELS models on a real EMA study dataset. We will also provide comparisons with existing methods such as MixRegLS [5] for MLE estimation, and brms [25] for MCMC estimation. All computations were run on a laptop equipped with a 14‐core Intel i7‐12700H CPU running at 4.70 GHz.

### 5.1 Simulations

To evaluate the performance of our proposed algorithm, we conducted three simulation studies. The first study assesses the algorithm's accuracy under a correctly specified model, the second examines its robustness when a key model assumption is violated, and the third study evaluates the consistency of our proposed estimator as the number of subjects and the observations per subject increase.

#### 5.1.1 Parameter Estimation Accuracy

In the first study, we evaluated the empirical properties of our VMP estimator. We generated 1000 datasets from the MELS model defined in Equation (2). Each simulated dataset contained N=500 subjects. To simulate an unbalanced longitudinal design, the number of observations per subject, $n_{i}$, was drawn from a discrete Uniform distribution U{20,21,…,40}.

The covariates were generated to mimic a typical longitudinal study. For each subject, we generated two time‐invariant covariates, a binary **group** indicator from a Ber(0.5), and a continuous variable $X_{1}$ from a 𝒩(5,2). We also generated two time‐varying covariates. The **time** variable was created by randomly sampling $n_{i}$ integer timepoints without replacement from the sequence {1,2,…,40}, and a second continuous variable is sampled from a 𝒩(3,1) distribution.

The covariates were then assigned to the different components of the MELS model:

(13a) $$\begin{array}{ll} & Y_{ij}=\beta_{0}+\beta_{1}\text{time}+\beta_{2}\text{group}+\beta_{3}X_{1}+\beta_{4}X_{4}+\nu_{i}+\epsilon_{ij}, \end{array}$$

(13b) $$\begin{array}{ll} & \log(\sigma_{\nu_{i}}^{2})=\alpha_{0}+\alpha_{1}\text{group}+\alpha_{2}X_{1}, \end{array}$$

(13c) $$\begin{array}{ll} & \log(\sigma_{\epsilon_{ij}}^{2})=\tau_{0}+\tau_{1}\text{time}+\tau_{2}X_{2}+\omega_{i}, \end{array}$$

(13d) $$\begin{array}{ll} & \beta ={\left(\beta_{0},\beta_{1},\beta_{2},\beta_{3},\beta_{4}\right)}^{⊤}={\left(15,-0.25,-2,0,0.5\right)}^{⊤}, \end{array}$$

(13e) $$\begin{array}{ll} & \alpha ={\left(\alpha_{0},\alpha_{1},\alpha_{2}\right)}^{⊤}={\left(1.2,0.6,-0.75\right)}^{⊤}, \end{array}$$

(13f) $$\begin{array}{ll} & \tau ={\left(\tau_{0},\tau_{1},\tau_{2}\right)}^{⊤}={\left(0.8,0.02,0\right)}^{⊤}, \end{array}$$

(13g) $$\begin{array}{ll} & \sigma_{\omega }=0.7; \end{array}$$

where 1 denotes the intercept. We then fitted our VMP algorithm to each of the 1000 datasets, yielding 1000 point estimates for each parameter. This collection of estimates forms an empirical sampling distribution for our VMP estimator. We assess the estimator's variability by calculating its 2.5% and 97.5% quantiles of this empirical distribution. If the means of the estimates align with the true value, it provides strong evidence that our estimator is unbiased and accurate. To supplement this analysis, we also calculated the empirical bias and Mean Squared Error (MSE) to further quantify the estimator's accuracy and precision.

Table 1 lists the simulation results of our VMP algorithm under a correctly specified model. The 95% empirical simulation intervals constructed from the 1000 replications successfully contain the true value for all of the estimated parameters $\left(\beta ,\alpha ,\tau ,\sigma_{\omega }\right)$. This provides strong evidence that our algorithm produces unbiased estimators. This is further supported by the negligible average bias and small MSE observed for all coefficients. Thus, the reliability of the algorithm for estimating both the scale and location parameters is confirmed.

**TABLE 1:** Simulation results for the VMP algorithm over 1000 replications.

Parameter	True value	95% empirical CI	Avg. bias	MSE
*Mean model parameters* (β)				
Intercept	15	(14.822, 15.162)	$-4\times 10^{-4}$	0.008
time	−0.25	(−0.252, −0.248)	$2\times 10^{-6}$	$1\times 10^{-6}$
group	−2	(−2.079, −1.915)	0.001	0.001
$X_{1}$	0	(−0.022, 0.025)	$3\times 10^{-4}$	$1\times 10^{-4}$
$X_{2}$	0.5	(0.474, 0.526)	$7\times 10^{-4}$	$2\times 10^{-4}$
*Between‐subject variance parameters* (α)				
Intercept	1.2	(0.659, 1.815)	0.025	0.085
group	0.6	(0.153, 1.071)	0.012	0.057
$X_{1}$	−0.75	(−0.918, −0.633)	−0.016	0.005
*Within‐subject variance parameters* (τ)				
Intercept	0.8	(0.673, 0.896)	−0.005	0.004
time	0.02	(0.018, 0.022)	$5\times 10^{-7}$	$1\times 10^{-6}$
$X_{2}$	0	(−0.024, 0.024)	$8\times 10^{-5}$	$1\times 10^{-4}$
$\sigma_{\omega }$	0.7	(0.648, 0.750)	$1\times 10^{-4}$	$6\times 10^{-4}$

*Note:* The table shows the true parameter values, the 95% empirical confidence interval of the estimates, the average bias, and the Mean Squared Error (MSE).

#### 5.1.2 Robustness to Correlated Random Effects

The second simulation study is designed to assess the robustness of our VMP algorithm to a direct violation of the mean field independence assumption. The WS variance model usually considers the correlation between the two random effects, with one formulation as [5]:

$$\exp(W_{ij}^{⊤}\tau +r\nu_{i}+\omega_{i}).$$

Here, r reflects the linear influence of $\nu_{i}$ on the WS variance, and represents the association of the subject's location effect with their WS variance. For this simulation, we generated data with r=−0.3, and all other parameter values remaining the same as in Equation (13). Then, our algorithm, which incorrectly assumes these effects are independent r=0, was fit to the data. Here, we consider the 95% empirical simulation interval, the average biases, and the MSE for our estimators.

Table 2 shows the simulation results under model mis‐specification. The key finding is that our VMP algorithm remains robust in terms of the primary fixed‐effect parameters (β,α,τ). The 95% empirical simulation intervals are still well‐centered and contain the true parameter values. Furthermore, the average biases and MSE remain small and comparable to the correctly specified model result presented in Table 1. This suggests that even when the independence assumption is violated, our algorithm can still provide accurate and reliable estimation for the main regression coefficients.

**TABLE 2:** Simulation results for the VMP algorithm under model misspecification where the random location and scale effects are correlated (r=−0.3).

Parameter	True value	95% empirical CI	Avg. bias	MSE
*Mean model parameters* (β)				
Intercept	15	(14.883, 15.229)	0.055	0.011
time	−0.25	(−0.252, −0.248)	$2\times 10^{-5}$	$1\times 10^{-6}$
group	−2	(−2.079, −1.916)	0.003	0.002
$X_{1}$	0	(−0.029, 0.019)	−0.004	$2\times 10^{-4}$
$X_{2}$	0.5	(0.473, 0.525)	$6\times 10^{-5}$	$2\times 10^{-4}$
*Between‐subject variance parameters* (α)				
Intercept	1.2	(0.614, 1.757)	$3\times 10^{-4}$	0.087
group	0.6	(0.166, 1.079)	0.015	0.058
$X_{1}$	−0.75	(−0.905, −0.628)	−0.014	0.005
*Within‐subject variance parameters* (τ)				
Intercept	0.8	(0.675, 0.912)	−0.005	0.004
time	0.02	(0.018, 0.022)	$-4\times 10^{-5}$	$1\times 10^{-6}$
$X_{2}$	0	(−0.025, 0.022)	$-5\times 10^{-4}$	$1\times 10^{-4}$
$\sigma_{\omega }$	0.7	(0.707, 0.815)	0.061	0.005

*Note:* The table shows the true parameter values, the 95% empirical confidence interval of the estimates, the average bias, and the Mean Squared Error (MSE) across 1000 replications.

However, the model mis‐specification had a predictable impact on the estimation of the standard deviation of the random scale effect $\sigma_{\omega }$. The result shows a noticeable positive average bias of 0.061, and the 95% empirical simulation interval (0.707,0.815) is shifted upward, failing to cover the true value of 0.7. This is an expected consequence due to the mean field approximation by forcing $q(\nu_{i})$ and $q(\omega_{i})$ to be independent; the algorithm inflates the variance of the scale random effect by absorbing the unaccounted dependence of the two random effects. Despite this, while the $\sigma_{\omega }$ parameter is often treated as a nuisance parameter in data analysis, the overall robustness of the fixed‐effect parameters highlights the practical utility even when the underlying assumptions are not perfectly met.

#### 5.1.3 Parameter Estimation Consistency

Beyond evaluating the finite sample properties, we conducted an empirical study to assess the consistency of our VMP estimator. Consistency, a fundamental asymptotic property, implies that as the sample size increases, the estimator converges to the true parameter value, leading to a decreasing Mean Squared Error (MSE) value as the sample size increases.

To investigate this, we varied both the number of subjects N and the observations per subject $n_{i}$. Specifically, we considered N∈{100,200,500} and $n_{i}=\{15,25,50,100,200\}$. For each combination of N and $n_{i}$, we generated 100 datasets with no missing time points following the parameter values in Equation (13), and fitted them with our VMP algorithm. The averaged MSE values and the corresponding standard deviation across all parameters were then calculated for each scenario.

**FIGURE 2:**
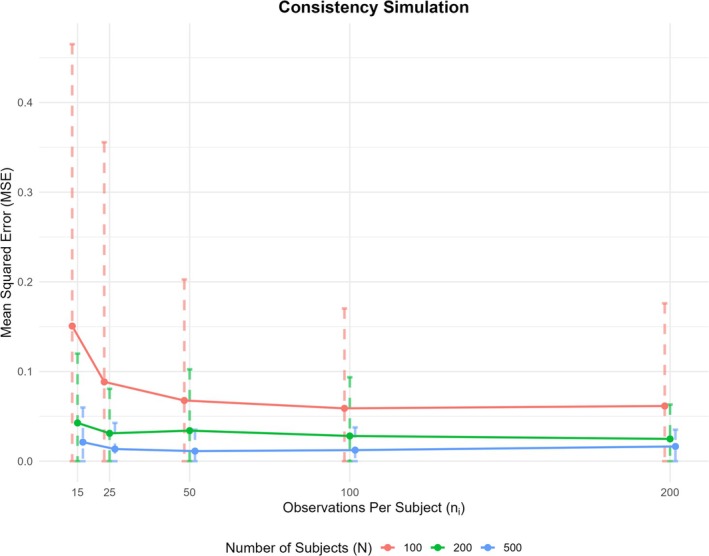
Consistency simulation results showing the Mean Squared Error (MSE) of the VMP estimator as a function of the number of observations per subject ($n_{i}$) for different numbers of subjects (N). Error bars represent the standard error of the MSE.

The results presented in Figure 2 clearly demonstrate the consistency of our VMP estimator. We can see that for all three different numbers of subjects N, the MSE decreases as $n_{i}$ increases. Similarly, for a fixed $n_{i}$, as the number of subjects N increases, the averaged MSE value also decreases. This suggests that consistency is achieved not only by the number of subjects N, but also by the observations per subject $n_{i}$. Moreover, we can see that the error bar, which is obtained by the ±2 standard deviations of the MSE estimates across replications (truncated at 0 since MSE cannot be negative), decreases as we increase N and $n_{i}$. This confirms that the MSE itself is estimated with greater precision in larger samples.

#### 5.1.4 Confidence Interval Coverage

We performed a simulation study to investigate the validity of confidence interval estimation provided by the Wald interval using the robust sandwich estimator and the non‐parametric bootstrap confidence interval. For this, we set the parameter values as in Equation (13), and performed 1000 simulations, constructing 95% confidence intervals using the original, uncorrected VMP estimated standard errors, the robust sandwich standard errors, and the non‐parametric bootstrap confidence quantiles. Then we compared the coverage and the ratio of the mean standard error (SE) of the two Wald intervals to the empirical standard errors of the 1000 replicated datasets. The results are provided in Table 3. Note that a well‐calibrated interval estimator should yield an SE ratio close to 1.0 and achieve a nominal coverage rate of approximately 95%. Also note that since the non‐parametric bootstrap uses the quantiles to construct confidence intervals, the SE ratio is not available.

**TABLE 3:** Average coverage and SE ratio by parameter and correction method.

Parameter	Variable	Average coverage			Average SE ratio	
		Unc.	Cor.	Boot.	Unc.	Cor.
Beta	Intercept	0.788	0.956	0.957	0.661	1.030
	Time	0.944	0.949	0.947	0.999	1.030
	Group	0.802	0.960	0.963	0.661	1.040
	X1	0.706	0.951	0.955	0.552	1.000
	X2	0.946	0.954	0.950	1.000	1.040
Alpha	Intercept	0.786	0.754	0.945	0.634	0.592
	Group	0.688	0.616	0.935	0.524	0.448
	X1	0.609	0.497	0.955	0.454	0.344
Tau	Intercept	0.873	0.951	0.962	0.745	0.966
	Time	0.943	0.954	0.954	0.968	1.010
	X2	0.932	0.951	0.950	0.948	1.000

Abbreviations: Boot. = Bootstrap, Cor. = Corrected (Sandwich), Unc. = Uncorrected.

As expected under the MFVI framework, the uncorrected VMP standard errors systematically underestimate the true empirical variation. Alternatively, the robust sandwich estimator successfully corrects this underestimation for all β and τ parameters. The corrected SE ratios map almost perfectly to the empirical standard deviations. Consequently, the sandwich intervals achieve the nominal 95% coverage rate for all β and τ parameters, proving to be a highly efficient, closed‐form solution for observation‐level inference.

Conversely, the analytical corrections break down for the between‐subject variance parameters α. Applying the sandwich estimator to α amplifies the underestimation, shrinking the SE ratios further and resulting in an even lower coverage rate than the uncorrected standard errors. This failure is a consequence of applying asymptotic analytical corrections to highly skewed, level‐2 variance components. While β and τ benefit from an effective sample size of $\sum_{i=1}^{N}n_{i}$, the α parameters are restricted to the subject‐level sample size (N), which may be insufficient for the asymptotic normality required of Wald intervals. Since analytical standard errors can fail for these specific variance components, the nonparametric bootstrap can resolve the under‐coverage issues for the α parameters. As shown in the table, the bootstrap restores coverage for all parameters around 95%. Ultimately, this demonstrates that leveraging the estimation speed of the VMP algorithm, which facilitates the use of the nonparametric bootstrap can provide a robust framework for inference in complex MELS models when asymptotic properties may fail.

#### 5.1.5 Computation Efficiency

A primary motivation for developing the MELS‐VMP algorithm is to overcome the computational bottlenecks associated with traditional estimation methods. To explicitly demonstrate this efficiency, we conducted a runtime comparison between the proposed algorithm, the non‐parametric bootstrap of our estimator, MLE by MixRegLS, and MCMC by brms.

For this simulation, the parameter values were kept the same as in Equation (13) and fixed the number of subjects at N=200, and gradually increased the number of observations per subject $n_{i}$ from 20 to 60. The models were fitted to each generated dataset, and the execution times were recorded.

The results, presented in Table 4, highlight a contrast in computational scalability. Our algorithm is remarkably fast and stable in terms of runtimes, and consistently converged in approximately 1 s regardless of the increase in observations per subject. While computationally more demanding by requiring about 5 min of computation time, the nonparametric bootstrap has advantages noted in the previous simulation study (i.e., it yields robust inference results without relying on strict distributional assumptions). The MLE method showed a steady increase in computation time as $n_{i}$ increased; it required nearly a minute and a half due to the growing burden of the adaptive Gauss‐Hermite quadrature evaluations. The MCMC approach was by far the most computationally demanding. While it took roughly 6 min for smaller datasets ($n_{i}=20$), the runtime scaled drastically, requiring almost an hour (3479 s) to fit a single model when $n_{i}=60$. This demonstrates that as the size of the dataset increases, MLE and MCMC become increasingly impractical for tasks that require repeated model fitting, such as cross‐validation, bootstrap, and model selection, whereas our algorithm handles intensive longitudinal data with negligible computational cost.

**TABLE 4:** Comparison of computation times (in seconds) for fitting the MELS model using VMP, MLE (MixRegLS), and MCMC (brms) across varying numbers of observations per subject ($n_{i}$).

Observations per subject ($n_{i}$)	VMP time (s)	Boot. time (s)	MLE time (s)	MCMC time (s)
20	0.97	321.12	22.73	350.07
30	0.70	295.20	29.91	467.48
40	0.60	331.27	44.31	1317.12
50	1.00	319.82	54.19	3176.33
60	1.08	329.45	88.39	3479.19

*Note:* The number of subjects is fixed at N=200.

### 5.2 Real Data

In this section, we will illustrate the ability of our model to perform statistical inference. For statistical inference, we usually rely on the assumption of normality. However, this assumption can easily be violated and produce problematic results. One way of doing robust statistical inference is to use the bootstrap. The traditional method of fitting MELS, such as MLE and MCMC, can be too time‐intensive for large datasets, making bootstrap impractical.

We will demonstrate the ability of our VMP algorithm on a real dataset from an EMA study on the effect of various covariates on positive mood variation in adolescents [33] with 516 subjects, 17 574 observations, and 17 covariates. The dataset contains the following outcomes and covariates:

- *posmood*: Prompt‐level assessment of the positive mood outcome.
- *t1, t2, t3, t4*: Time‐of‐day indicators of 9 am–1:59 pm, 2 pm–5:59 pm, 6 pm–9:59 pm, and 10 pm–2:59 am, leaving 3 am–8:59 pm as the reference time indicator.
- *w1, w2, w3, w4, w5, w6*: Day‐of‐week indicators of Monday, Tuesday, …, Saturday, leaving Sunday as the reference day indicator.
- *other_bs*: Subject mean of the prompt‐varying “with others” indicator (proportion of prompts in which a subject was with others.)
- *other_ws*: Prompt‐varying deviation of the “with others” indicator minus the subject mean of this variable.
- *genderf*: Coded 0 for males and 1 for females.
- *age15*: The subject's age minus 15.
- *tirbor*: Prompt‐level assessments of tired/bored.
- *frustr*: Prompt‐level assessments of frustrated/stressed.

We applied our VMP algorithm to the EMA study dataset above to model the positive mood of subjects, with the goal of demonstrating both parameter estimation and statistical inference. We compare our VMP algorithm against two established methods: A standard MLE approach implemented in MixRegLS and a full Bayesian analysis using Hamiltonian Markov Chain implemented in brms. For the Bayesian approach, the priors are specified as: $\beta ∼𝒩(0,10^{4})$, $\alpha ∼𝒩(0,10^{4})$, $\tau ∼𝒩(0,10^{4})$ and $\sigma_{\omega }^{2}∼\text{Half‐Cauchy}(10^{4})$, running with four chains and 2000 iterations each where the first 1000 are used for warm up and the following 1000 are used for posterior inference. For bootstrap, 1000 replications were used that were parallelized into 10 CPU cores. The model specification is as follows:

$$\begin{array}{ll} & Y_{ij}=\beta_{0}+\beta_{1}\text{other\_}\text{bs}+\beta_{2}\text{other\_}\text{ws}+\beta_{3}\text{genderf}+\beta_{4}\text{t1}\\ & \;+\beta_{5}\text{t2}+\beta_{6}\text{t3}+\beta_{7}\text{t4}+\beta_{8}\text{w1}+\beta_{9}\text{w2}+\beta_{10}\text{w3}\\ & \;+\beta_{11}\text{w4}+\beta_{12}\text{w5}+\beta_{13}\text{w6}+\beta_{14}\text{tirbor}+\beta_{15}\text{frustr}+\nu_{i}+\epsilon_{ij},\\ & \log(\sigma_{\nu_{i}}^{2})=\alpha_{0}+\alpha_{1}\text{other\_}\text{bs}+\alpha_{2}\text{genderf}+\alpha_{3}\text{age15},\\ & \log(\sigma_{\epsilon_{ij}}^{2})=\tau_{0}+\tau_{1}\text{other\_}\text{bs}+\tau_{2}\text{other\_}\text{ws}+\tau_{3}\text{genderf}\\ & \;+\tau_{4}\text{age15}+\tau_{5}\text{tirbor}+\tau_{6}\text{frustr}+\omega_{i};\\ & \beta ={\left(\beta_{0},\beta_{1},\ldots ,\beta_{15}\right)}^{⊤},\\ & \alpha ={\left(\alpha_{0}.\alpha_{1}.\alpha_{2},\alpha_{3}\right)}^{⊤},\\ & \tau ={\left(\tau_{0},\tau_{1},\ldots ,\tau_{6}\right)}^{⊤}, \end{array}$$

The analysis results are presented in Table 5, which compares the point estimates and the interval estimates obtained from our VMP algorithm, MLE, and MCMC. The VMP bootstrap intervals were generated by sampling subjects with replacement.

**TABLE 5:** Parameter estimates and 95% interval estimates from the real data analysis of the EMA smoking study.

Param.	VMP	VMP Wald	VMP boot.	MLE	MLE Wald	MCMC	MCMC C.I.
*Mean model parameters* (β)							
Intercept	8.143	(7.621, 8.666)	(7.629, 8.692)	8.138	(7.662, 8.615)	8.155	(7.664, 8.642)
other_bs	0.462	(−0.178, 1.101)	(−0.172, 1.115)	0.456	(−0.161, 1.074)	0.444	(−0.175, 1.064)
other_ws	0.191	(0.135, 0.248)	(0.136, 0.248)	0.191	(0.143, 0.239)	0.191	(0.143, 0.237)
genderf	−0.029	(−0.214, 0.156)	(−0.226, 0.153)	−0.028	(−0.218, 0.162)	−0.050	(−0.244, 0.144)
t1	0.247	(0.173, 0.322)	(0.175, 0.320)	0.247	(0.179, 0.315)	0.247	(0.178, 0.317)
t2	0.355	(0.271, 0.439)	(0.274, 0.432)	0.355	(0.286, 0.424)	0.355	(0.284, 0.423)
t3	0.384	(0.304, 0.464)	(0.304, 0.458)	0.384	(0.315, 0.453)	0.384	(0.313, 0.454)
t4	0.300	(0.184, 0.416)	(0.183, 0.408)	0.300	(0.201, 0.399)	0.299	(0.198, 0.400)
w1	0.140	(0.050, 0.230)	(0.053, 0.233)	0.140	(0.070, 0.211)	0.140	(0.069, 0.211)
w2	0.169	(0.067, 0.270)	(0.068, 0.271)	0.169	(0.098, 0.239)	0.169	(0.096, 0.240)
w3	0.128	(0.025, 0.231)	(0.024, 0.234)	0.128	(0.057, 0.199)	0.127	(0.058, 0.199)
w4	0.082	(−0.011, 0.175)	(−0.009, 0.178)	0.082	(0.014, 0.150)	0.081	(0.015, 0.149)
w5	0.050	(−0.034, 0.134)	(−0.035, 0.136)	0.050	(−0.021, 0.121)	0.049	(−0.020, 0.122)
w6	−0.022	(−0.101, 0.058)	(−0.101, 0.056)	−0.022	(−0.093, 0.049)	−0.023	(−0.090, 0.047)
tirbor	−0.146	(−0.162, −0.130)	(−0.162, −0.131)	−0.146	(−0.156, −0.135)	−0.146	(−0.156, −0.135)
frustr	−0.326	(−0.344, −0.308)	(−0.345, −0.307)	−0.326	(−0.338, −0.315)	−0.326	(−0.338, −0.315)
*Between‐subject variance parameters* (α)							
Intercept	−0.188	(−0.873, 0.498)	(−0.936, 0.488)	−0.199	(−0.854, 0.455)	−0.162	(−0.796, 0.481)
other_bs	0.332	(−0.604, 1.268)	(−0.628, 1.257)	0.330	(−0.545, 1.205)	0.300	(−0.576, 1.134)
genderf	0.093	(−0.193, 0.378)	(−0.182, 0.392)	0.094	(−0.176, 0.364)	0.091	(−0.177, 0.347)
age15	−0.104	(−0.232, 0.024)	(−0.232, 0.027)	−0.107	(−0.229, 0.014)	−0.104	(−0.219, 0.012)
*Within‐subject variance parameters* (τ)							
Intercept	0.193	(−0.142, 0.528)	(−0.105, 0.579)	0.219	(−0.081, 0.518)	0.212	(−0.086, 0.524)
other_bs	−0.306	(−0.712, 0.101)	(−0.697, 0.108)	−0.285	(−0.665, 0.096)	−0.280	(−0.674, 0.102)
other_ws	−0.092	(−0.160, −0.024)	(−0.167, −0.024)	−0.092	(−0.148, −0.036)	−0.092	(−0.148, −0.034)
genderf	0.168	(0.055, 0.281)	(0.056, 0.282)	0.175	(0.062, 0.288)	0.174	(0.062, 0.292)
age15	−0.085	(−0.136, −0.034)	(−0.134, −0.032)	−0.083	(−0.134, −0.032)	−0.082	(−0.132, −0.032)
tirbor	−0.004	(−0.020, 0.011)	(−0.021, 0.012)	−0.004	(−0.015, 0.007)	−0.004	(−0.016, 0.006)
frustr	0.130	(0.115, 0.145)	(0.114, 0.146)	0.130	(0.119, 0.140)	0.130	(0.120, 0.140)
$\sigma_{\omega }$	0.588	NA	(0.541, 0.628)	0.587	(0.543, 0.631)	0.592	(0.546, 0.640)

*Note:* The table presents point estimates from the VMP algorithm, MLE, and MCMC, alongside four interval types: The VMP Wald robust confidence interval, the VMP bootstrap confidence interval, the MLE Wald confidence interval, and the MCMC credible interval.

#### 5.2.1 Point Estimation

The first key finding from Table 5 is the remarkable agreement between the point estimates from our VMP algorithm and those from MLE. Across all results for β, α, τ and $\sigma_{\omega }$, the estimates are near identical. This shows that our algorithm successfully converges to the same optimal parameter values as the established, computationally intensive MLE method, confirming the accuracy in terms of point estimation of our estimators.

#### 5.2.2 Interval Estimation and Inference

A comparison of the confidence and credible intervals reveals important insights into the performance of different approaches. The VMP Wald intervals obtained by the robust standard errors from Section 4.3 are comparable with the results obtained by the MLE and MCMC methods, validating the use of these estimates for statistical inference. The VMP bootstrap intervals use the naive empirical bootstrap [29], which could work better for complex model settings compared to bootstrap‐t, and generally align well with the MLE Wald intervals and MCMC credible intervals. These results suggest that applying an ad‐hoc sandwich estimator or bootstrap procedure to our fast VMP algorithm corrects the underestimation of variance due to the mean field approximation. This provides a reliable interval estimation that is comparable with both the MLE and MCMC approaches.

Based on the interval estimates in Table 5, our analysis yields several insights into positive mood dynamics. For the average positive mood (location effect), we found that subject levels of momentary socializing (other_bs) and occasion‐specific momentary socializing (other_ws) significantly increases positive mood. Alternatively, feeling tired/bored and frustrated/stressed significantly decreased levels of positive mood. A key advantage of the MELS model is the ability to analyze mood volatility (i.e., within‐subject scale effect). Our results show that momentary socializing and older age are associated with significantly more stable moods (lower variance). Conversely, feeling frustrated and identifying as female are associated with significantly greater mood volatility. None of the tested covariates were significant predictor of the between‐subject variance.

#### 5.2.3 Computation Time

Beyond statistical accuracy, a key advantage of our VMP algorithm is its computational efficacy, especially when compared to MLE and MCMC methods. As shown in Table 6, the standard VMP algorithm converged remarkably quickly, taking approximately 1.5 s on the EMA study dataset. As expected, the MCMC approach using brms was significantly more time‐consuming, requiring approximately 1.5 h for a 2000 iterations chain to be sampled for stable posterior inference. The MLE approach via MIXRegLS, which relies on numerical integration, took approximately 5 min.

**TABLE 6:** Comparison of approximate computational times for fitting the MELS model to the EMA dataset using different estimation and inference approaches.

Approach	VMP	VMP bootstrap	MLE	MCMC
Time	≈1.5 s	≈9 min	≈4 min	≈1.5 h

The VMP bootstrap procedure, while adding computational overhead to the base VMP fit, remained efficient relative to MCMC, taking approximately 9 min. This shows that the VMP bootstrap method offers substantial time savings compared to MCMC and is competitive with MLE. Critically, bootstrapping retains a key advantage often associated with MCMC: Robustness to non‐normality. Unlike standard MLE Wald intervals, which rely on asymptotic normality assumptions, both MCMC credible intervals and bootstrap confidence intervals provide more reliable inference when estimator distributions deviate from normality. Furthermore, the ability to parallelize the bootstrap procedure enhances its efficiency on multi‐core processors. This combination of speed and robustness makes VMP well‐suited to iterative tasks such as model selection on large datasets, where fitting the MELS models multiple times with MLE or MCMC can be slow or lack robustness.

## 6 Discussion

Fitting MELS models to longitudinal datasets has traditionally forced a trade‐off between statistical precision and computational feasibility. Standard methods such as MLE and MCMC, while robust, are often too computationally demanding for iterative tasks such as model selection for large datasets. In this paper, we introduce the MELS‐VMP algorithm to take advantage of the speed of VI for fast parameter estimation without sacrificing inference validity. We propose the whole algorithm for fitting MELS models while bridging the gap between VI's computational efficiency and the need for valid statistical inference results. This combination of speed and reliability makes complex tasks such as exploratory data analysis and model selection computationally practical, even when faced with large, intensive longitudinal datasets. As a practical contribution, we have also developed an R package that implements the proposed MELS‐VMP algorithm, which includes the dataset used for our demonstration in Section 5.2. This package is publicly available at https://github.com/BrianWu06/melsvmp.

While the primary focus of this paper is on parameter estimation and inference, the computational efficiency of MELS‐VMP makes it highly appealing for model selection of fixed effects. To perform variable selection, we can rely on established variational information criteria and *p*‐values obtained via robust standard errors. Specifically, we can compute the Variational Bayesian Information Criterion (VBIC) and the Variational Akaike Information Criterion (VAIC) [34]. The VBIC leverages the converged ELBO to consistently identify the true model, while the VAIC approximates the Deviance Information Criterion to select models optimized for predictive accuracy. Because these criteria can be obtained directly from the converged variational parameter estimates, they allow for instant screening of candidate models. Additionally, since *p*‐values can be derived from the robust standard errors under the profile M‐estimator framework, multiple testing corrections such as the Benjamini‐Hochberg procedure [35] can also be effectively utilized for model selection.

It is also important to note that we do not propose MELS‐VMP as a replacement for traditional methods but rather as a powerful and essential complement. As suggested [9] in the paper that introduced VI, it is a powerful tool for large‐scale data analysis when other time‐consuming algorithms are running. We recommend an efficient workflow where analysts first use the fast MELS‐VMP algorithm for initial exploration and to rapidly screen through a large set of candidate models. Once the set is narrowed down, researchers can then invest the necessary computational time to fit the final, promising models using MCMC or MLE, which rely on fewer numerical approximations.

Finally, the VMP framework can be used to provide extension beyond the foundational MELS model discussed in this paper. For instance, our approach can be extended to more complex hierarchical structures, such as the 3‐level MELS model [21]. This flexibility is valuable since VI sees growing popularity in quantitative psychology for models such as Item Response Theory [36] or Structural Equation Models [37]. Furthermore, emerging frameworks built on VI, such as amortized inference [38, 39], offer possibilities to tackle even more intractable models while retaining VI's key advantage of computational speed.

## Funding

This work was supported by the National Institutes of Health (Grant No. R01CA240713).

## Conflicts of Interest

The authors declare no conflicts of interest.

## Supporting information


**Data S1**: sim70640‐sup‐0001‐Supinfo.pdf.

## Data Availability

The data that support the findings of this study are available on request from the corresponding author. The data are not publicly available due to privacy or ethical restrictions.
