# Supplementary material for: Fast Estimation and Valid Statistical Inference for Mixed‐Effect Location‐Scale Models Using Variational Inference
Source: Stat Med. 2026 Jun 17;45(13-14):e70640. doi: 10.1002/sim.70640 (PMC13276028; doi:10.1002/sim.70640)
Supplement: Supplementary file 1 — Data S1: sim70640‐sup‐0001‐Supinfo.pdf. [file SIM-45-0-s001.pdf]

Supplementary file for "Fast Estimation and Valid Statistical Inference for Mixed-Effect Location-Scale Models Using Variational Inference"

A Derivation of Variational Updates

This section provides the whole derivation for the updates of all parameters in our MELS-VMP algorithm. For simplicity, we will define the following expectation terms:

$$
\begin{aligned}
h_{ij} &= \mathbb{E}_q[(y_{ij} - X_{ij}^\top \beta - \nu_i)^2] = (y_{ij} - X_{ij}^\top \mu_\beta^q - \mu_{\nu_i}^q)^2 + X_{ij}^\top \Sigma_\beta^q X_{ij} + \Sigma_{\nu_i}^q \\
\psi_{ij}^\tau &= \mathbb{E}_q[\exp(-W_{ij}^\top \tau)] = \exp(-W_{ij}^\tau \mu_\tau^q + \frac{1}{2} W_{ij}^\top \Sigma_\tau^q W_{ij}) \\
\psi_i^\omega &= \mathbb{E}_q[\exp(-\omega_i)] = \exp(-\mu_{\omega_i}^q + \frac{1}{2}\Sigma_{\omega_i}^q) \\
\psi_i^\alpha &= \mathbb{E}_q[\exp(-U_i^\top \alpha)] = \exp(-U_i^\top \mu_\alpha^q + \frac{1}{2} U_i^\top \Sigma_\alpha^q U_i)
\end{aligned}
$$

A.1 Update for β parameters

While β is conjugate in our model, we can have the update rule:

$$\log q^*(\beta) = \mathbb{E}_{q-\beta}[\log p(y, \theta)] + C$$

with C as a constant. Since we only need the terms that involve β, this can be further simplified as

$$\log q^*(\beta) = \mathbb{E}_{q-\beta}[\log p(\beta) + \sum_{i,j} \log p(y_{ij} \mid \beta, \tau, \nu_i, \omega_i)] + C$$

We have:

$$\log p(\beta) = \frac{-1}{2\sigma_\beta^2} \beta^\top \beta + C$$

$$\log p(y_{ij} \mid \dots) = -\frac{1}{2}(y_{ij} - X_{ij}^\top \beta - \nu_i)^2 \exp(-W_{ij}^\top \tau + \omega_i) + C$$

By combining and taking the expectation to these two terms, we can get:

$$\begin{aligned}
\log q^*(\beta) &= \frac{-1}{2\sigma_\beta^2}\beta^\top\beta - \frac{1}{2}\mathbb{E}_{q-\beta}[\sum_{i,j}(y_{ij} - X_{ij}^\top\beta - \nu_i)^2\exp(-W_{ij}^\top\tau + \omega_i)] + C \\
&= \frac{-1}{2\sigma_\beta^2}\beta^\top\beta - \frac{1}{2}\sum_{i,j}\psi_{ij}^\tau\psi_i^\omega[(y_{ij} - X_{ij}^\top\beta - \mu_{\nu_i}^q)^2 - \Sigma_{\nu_i}^q] + C \\
&= \frac{-1}{2\sigma_\beta^2}\beta^\top\beta - \frac{1}{2}\sum_{i,j}\psi_{ij}^\tau\psi_i^\omega[\beta^\top X_{ij}X_{ij}^\top\beta - 2\beta^\top X_{ij}(y_{ij} - \mu_{\nu_i}^q)] + C \\
&= \frac{-1}{2}\beta^\top(\frac{1}{\sigma_\beta^2}\mathcal{I} + \sum_{i,j}\psi_{ij}^\tau\psi_i^\omega X_{ij}X_{ij}^\top)\beta + \beta^\top[\sum_{i,j}\psi_{ij}^\tau\psi_i^\omega(y_{ij} - \mu_{\nu_i}^q)X_{ij}] + C
\end{aligned}$$

Since a normal distribution $\mathcal{N}(\mu, \Sigma)$ has log-density

$$-\frac{1}{2}\beta^\top\Sigma^{-1}\beta + \beta^\top\Sigma^{-1}\mu + c$$

we can match the log-densities and get

$$\begin{aligned}
\Sigma_\beta^q &\leftarrow (\frac{1}{\sigma_\beta^2}\mathcal{I} + \sum_{i,j}\psi_{ij}^\tau\psi_i^\omega X_{ij}X_{ij}^\top)^{-1} \\
\mu_\beta^q &\leftarrow \Sigma_\beta^q[\sum_{i,j}\psi_{ij}^\tau\psi_i^\omega(y_{ij} - \mu_{\nu_i}^q)X_{ij}]
\end{aligned}$$

A.2 Update for ν_i parameters

Similarly to β, ν_i's also have the Normal-Normal conjugacy property that allows us to obtain simple closed-form updates.

$$\log q^*(\nu_i) = \mathbb{E}_{q-\nu_i}[\log p(\nu_i \mid \alpha) + \sum_j \log p(y_{ij} \mid \dots)] + C$$

We have:

$$\begin{aligned}
\log p(\nu_i \mid \alpha) &= \frac{1}{2}\nu_i^2\exp(-U_i^\top\alpha) + C \\
\log p(y_{ij} \mid \dots) &= \frac{-1}{2}(y_{ij} - X_{ij}^\top\beta - \nu_i)^2\exp(-W_{ij}^\top\tau + \omega_i) + C
\end{aligned}$$

By combining and taking the expectation to these two terms, we can get:

$$
\begin{aligned}
\log q^*(\nu_i) &= \frac{-1}{2}\mathbb{E}_{q-\nu_i}[U_i^\top \alpha + \nu_i^2 \exp(-U_i^\top \alpha) + \sum_j (y_{ij} - X_{ij}^\top \beta - \nu_i)^2 \exp(-W_{ij}^\top \tau + \omega_i)] + C \\
&= \frac{-1}{2}\psi_i^\alpha \nu_i^2 - \frac{1}{2}\sum_j \psi_{ij}^\tau \psi_i^\omega (y_{ij} - X_{ij}^\top \mu_\beta^q - \nu_i)^2 + C \\
&= \frac{-1}{2}\psi_i^\alpha \nu_i^2 - \frac{1}{2}\sum_j \psi_{ij}^\tau \psi_i^\omega [-2(y_{ij} - X_{ij}^\top \mu_\beta^q)\nu_i + \nu_i^2] + C \\
&= \frac{-1}{2}(\psi_i^\alpha + \sum_j \psi_{ij}^\tau \psi_i^\omega)\nu_i^2 + \sum_j \psi_{ij}^\tau \psi_i^\omega (y_{ij} - X_{ij}^\top \mu_\beta^q)\nu_i + C
\end{aligned}
$$

Then by matching the log-density of normal distribution:

$$
\begin{aligned}
\Sigma_{\nu_i}^q &\leftarrow (\psi_i^\alpha + \sum_j \psi_{ij}^\tau \psi_i^\omega)^{-1} \\
\mu_{\nu_i}^q &\leftarrow \Sigma_{\nu_i}^q [\sum_j \psi_{ij}^\tau \psi_i^\omega (y_{ij} - X_{ij}^\top \mu_\beta^q)]
\end{aligned}
$$

A.3 Update for α parameters

For α, since the variance component is linked by an exponential function, the Normal-Normal conjugacy no longer exists, and we will need to turn to the simplified Laplace approximation in Equation **??**.

$$
L(\alpha) = \mathbb{E}_q[\log p(\alpha) + \sum_i \log p(\nu_i \mid \alpha)]
$$

The log-densities are:

$$
\begin{aligned}
\log p(\alpha) &= \frac{-1}{2\sigma_\alpha^2}\alpha^\top \alpha + C \\
\log p(\nu_i \mid \alpha) &= \frac{-1}{2}U_i^\top \alpha - \frac{-1}{2}\nu_i^2 \exp(-U_i^\top \alpha) + C
\end{aligned}
$$

By combining and taking expectations, we can get:

$$
L(\alpha) = \frac{-1}{2\sigma_\alpha^2}\mu_\alpha^{q\top}\mu_\alpha^q - \frac{1}{2}[U_i^\top \mu_\alpha^q + \psi_i^\alpha({\mu_{\nu_i}^q}^2 + \Sigma_{\nu_i}^q)] + C
$$

Next we take the gradient and Hessian with respect to μ_α^q

$$
\begin{aligned}
D_{\mu_\alpha^q} L(\alpha) &= \frac{-1}{\sigma_\alpha^2}\mu_\alpha^q + \frac{1}{2}\sum_i [\psi_i^\alpha({\mu_{\nu_i}^q}^2 + \Sigma_{\nu_i}^q) - 1]U_i \\
H_{\mu_\alpha^q} L(\alpha) &= \frac{-1}{\sigma_\alpha^2}\mathcal{I} - \frac{1}{2}\sum_i \psi_i^\alpha({\mu_{\nu_i}^q}^2 + \Sigma_{\nu_i}^q)U_i U_i^\top
\end{aligned}
$$

Then the updates for α can be obtained by the general update rule

$$\Sigma_\alpha^q \leftarrow [\frac{1}{\sigma_\alpha^2}\mathcal{I} + \frac{1}{2}\sum_i \psi_i^\alpha ({\mu_{\nu_i}^q}^2 + \Sigma_{\nu_i}^q) U_i U_i^\top]^{-1}$$
$$\mu_\alpha^q \leftarrow \mu_\alpha^q + \Sigma_\alpha^q \{\frac{-1}{\sigma_\alpha^2}\mu_\alpha^q + \frac{1}{2}\sum_i [\psi_i^\alpha ({\mu_{\nu_i}^q}^2 + \Sigma_{\nu_i}^q) - 1] U_i\}^\top$$

A.4 Update for τ parameters

Next we will derive the variational update for τ, which is similar to α that needs to be derived by the simplifies Laplace approximation.

$$L(\tau) = \mathbb{E}_q[\log p(\tau) + \sum_{i,j} \log p(y_{ij} \mid \dots)]$$

The log-densities are:

$$\log p(\tau) = \frac{-1}{2\sigma_\tau^2}\tau^\top \tau + C$$
$$\log p(y_{ij} \mid \dots) = \frac{-1}{2} W_{ij}^\top \tau - \frac{1}{2}(y_{ij} - X_{ij}^\top \beta - \nu_i)^2 \exp(-W_{ij}^\top \tau) + C$$

By combining and taking expectation, we can get:

$$L(\tau) = \frac{-1}{2\sigma_\tau^2}{\mu_\tau^q}^\top \mu_\tau^q - \frac{1}{2}\sum_{i,j}(W_{ij}^\top \mu_\tau^q + \psi_{ij}^\tau \psi_i^\omega h_{ij}) + C$$

Next we take the gradient and Hessian with respect to μ_τ^q

$$D_{\mu_\tau^q} L(\tau) = \frac{-1}{\sigma_\tau^2}\mu_\tau^q + \frac{1}{2}\sum_{i,j}(\psi_{ij}^\tau \psi_i^\omega h_{ij} - 1) W_{ij}$$
$$H_{\mu_\tau^q} L(\tau) = \frac{-1}{\sigma_\tau^2}\mathcal{I} - \frac{1}{2}\sum_{i,j} \psi_{ij}^\tau \psi_i^\omega h_{ij} W_{ij} W_{ij}^\top$$

The updates for τ can then be obtained

$$\Sigma_\tau^q \leftarrow (\frac{1}{\sigma_\tau^2}\mathcal{I} + \frac{1}{2}\sum_{i,j} \psi_{ij}^\tau \psi_i^\omega h_{ij} W_{ij} W_{ij}^\top)^{-1}$$
$$\mu_\tau^q \leftarrow \mu_\tau^q + \Sigma_\tau^q [\frac{-1}{\sigma_\tau^2}\mu_\tau^q + \frac{1}{2}\sum_{i,j}(\psi_{ij}^\tau \psi_i^\omega h_{ij} - 1) W_{ij}]^\top$$

A.5 Update for ω_i parameters

The final variable that their parameters need to be derived by the simplified Laplace approximations are the ω_i's.

$$L(\omega_i) = \mathbb{E}_q[\log p(\omega_i \mid \sigma_\omega^2) + \sum_j \log p(y_{ij} \mid \dots)]$$

The log-densities are:

$$\log p(\omega_i \mid \sigma_\omega^2) = \frac{-1}{2}\frac{\omega_i^2}{\sigma_\omega^2}$$
$$\log p(y_{ij} \mid \dots) = \frac{-1}{2}\omega_i - \frac{1}{2}(y_{ij} - X_{ij}^\top \beta - \nu_i)^2 \exp(-\omega_i) + C$$

By combining and taking expectation, we can get:

$$L(\omega_i) = \frac{-1}{2}{\mu_{\omega_i}^q}^2 \frac{A_{\sigma_\omega^2}^q}{B_{\sigma_\omega^2}^q} - \frac{1}{2}\sum_j (\mu_{\omega_i}^q + \psi_{ij}^\tau \psi_i^\omega h_{ij}) + C$$

Next we take the gradients and Hessians with respect to $\mu_{\omega_i}^q$:

$$D_{\mu_{\omega_i}^q} L(\omega_i) = -\mu_{\omega_i}^q \frac{A_{\sigma_\omega^2}^q}{B_{\sigma_\omega^2}^q} + \frac{1}{2}\sum_j (\psi_{ij}^\tau \psi_i^\omega h_{ij} - 1)$$
$$H_{\mu_{\omega_i}^q} L(\omega_i) = -\frac{A_{\sigma_\omega^2}^q}{B_{\sigma_\omega^2}^q} - \frac{1}{2}\sum_j \psi_{ij}^\tau \psi_i^\omega h_{ij}$$

The updates for ω_i can then be obtained

$$\Sigma_{\omega_i}^q \leftarrow (\frac{A_{\sigma_\omega^2}^q}{B_{\sigma_\omega^2}^q} + \frac{1}{2}\sum_j \psi_{ij}^\tau \psi_i^\omega h_{ij})^{-1}$$
$$\mu_{\omega_i}^q \leftarrow \mu_{\omega_i}^q + \Sigma_{\omega_i}^q[-\mu_{\omega_i}^q \frac{A_{\sigma_\omega^2}^q}{B_{\sigma_\omega^2}^q} + \frac{1}{2}\sum_j (\psi_{ij}^\tau \psi_i^\omega h_{ij} - 1)]$$

A.6 Update for σ_ω^2 parameters

σ_ω^2 parameters have a clean, conjugate update rule due to the Inverse-Gamma-Inverse-Gamma conjugacy.

$$\log q^*(\sigma_\omega^2) = \mathbb{E}_q[\log p(\sigma_\omega^2 \mid a_\omega) + \sum_i \log p(\omega_i \mid \sigma_\omega^2)] + C$$

Since $\sigma_\omega^2 \sim \text{I.G.}(1/2, 1/a_\omega)$, the log-densities are:

$$\log p(\sigma_\omega^2 \mid a_\omega) = \frac{-3}{2}\log \sigma_\omega^2 - \frac{1}{\sigma_\omega^2}\frac{1}{a_\omega} + C$$
$$\log p(\omega_i \mid \sigma_\omega^2) = \frac{-1}{2}\log \sigma_\omega^2 - \frac{\omega_i^2}{2\sigma_\omega^2} + C$$

By combining and taking the expectation, we can get:

$$\log q^*(\sigma_\omega^2) = \mathbb{E}_{q-\sigma_\omega^2}[\frac{-3}{2}\log \sigma_\omega^2 - \frac{1}{\sigma_\omega^2}\frac{1}{a_\omega} + \sum_i (\frac{-1}{2}\log \sigma_\omega^2 - \frac{\omega_i^2}{2\sigma_\omega^2})] + C$$
$$= -\frac{N+3}{2}\log \sigma_\omega^2 - \frac{1}{\sigma_\omega^2}[\frac{A_{a_\omega}^q}{B_{a_\omega}^q} + \sum_i ({\mu_{\omega_i}^q}^2 + \Sigma_{\omega_i}^q)] + C$$

Since an Inverse-Gamma$(x; A, B)$ has log-density

$$-(A+1)\log x - \frac{B}{x} + C$$

We can match the log-densities and get:

$$A^q_{\sigma^2_\omega} \leftarrow \frac{N+1}{2}$$
$$B^q_{\sigma^2_\omega} \leftarrow \frac{A^q_{a_\omega}}{B^q_{a_\omega}} + \sum_i ({\mu^q_{\omega_i}}^2 + \Sigma^q_{\omega_i})$$

A.7 Update for a_ω parameters

The final parameter update to derive is a_ω, which is also an Inverse-Gamma distribution and has a similar update derivation as σ^2_ω.

$$\log q^*(a_\omega) = \mathbb{E}_q[\log p(a_\omega) + \log p(\sigma^2_\omega \mid a_\omega)]$$

The log-densities are:

$$\log p(a_\omega) = -\frac{3}{2}\log a_\omega - \frac{1}{A_\omega a_\omega} + C$$
$$\log p(\sigma^2_\omega \mid a_\omega) = \frac{-1}{2}\log a_\omega - \frac{1}{a_\omega \sigma^2_\omega}$$

By combining and taking the expectation, we can get:

$$\begin{aligned}\log q^*(a_\omega) &= \mathbb{E}_{q-a_\omega}[-\frac{3}{2}\log a_\omega - \frac{1}{A_\omega a_\omega} - \frac{1}{2}\log a_\omega - \frac{1}{a_\omega \sigma^2_\omega}] + C \\ &= -2\log a_\omega - \frac{1}{a_\omega}(\frac{A^q_{\sigma^2_\omega}}{B^q_{\sigma^2_\omega}} + \frac{1}{A_\omega}) + C\end{aligned}$$

Then we can match the log-densities of Inverse-Gamma:

$$A^q_{a_\omega} \leftarrow 1$$
$$B^q_{a_\omega} \leftarrow \frac{A^q_{\sigma^2_\omega}}{B^q_{\sigma^2_\omega}} + \frac{1}{A_\omega}$$

B Derivation of the sandwich estimator

This appendix details the derivation of the robust sandwich estimator described in Section **??**. The key components are defined as follows:

Global parameters θ: The vector of global mean parameters we seek to make inference on, which is $\theta = (\mu^q_\beta, \mu^q_\alpha, \mu^q_\tau)$.

Local parameters ζ_i: The vector of local mean parameters for subject i, defined as $\zeta_i = (\mu^q_{\nu_i}, \mu^q_{\omega_i})$.

Per-subject ELBO L_i: The function $L_i(\theta, \zeta_i)$ is the sum of all terms inside the ELBO that depend on the data for subject i or the local parameters ζ_i. We ignore the local entropy terms $\mathbb{E}_q[\log q(\nu_i)]$ and $\mathbb{E}_q[\log q(\omega_i)]$ since they do not depend on the parameters in interest θ.

$$
\begin{aligned}
L_i &= \mathbb{E}_q[\log p(\nu_i \mid \alpha)] + \mathbb{E}_q[\log p(\omega_i \mid \sigma^2_\omega)] + \sum_j \mathbb{E}_q[\log p(y_{ij} \mid \dots)] \\
&= \frac{-1}{2}[U_i^\top \mu^q_\alpha + \psi^\alpha_i({\mu^q_{\nu_i}}^2 + \Sigma^q_{\nu_i})] - \frac{1}{2}({\mu^q_{\omega_i}}^2 + \Sigma^q_{\omega_i})\frac{A^q_{\sigma^2_\omega}}{B^q_{\sigma^2_\omega}} - \frac{1}{2}\sum_j [W_{ij}^\top \mu^q_\tau + \mu^q_{\omega_i} + \psi^\tau_{ij}\psi^\omega_i h_{ij}]
\end{aligned}
$$

B.1 The Meat Matrix $\hat{B}$

The meat matrix is $\hat{B} = \sum_i \hat{G}_i \hat{G}_i^\top$, and the core component $\hat{G}_i$ is defined as

$$\hat{G}_i = [\frac{\partial L_i}{\partial \mu^q_\beta}, \frac{\partial L_i}{\partial \mu^q_\alpha}, \frac{\partial L_i}{\partial \mu^q_\tau}]$$

These are the i-th subject contribution to the gradients we derived earlier, without the prior terms.

$$
\begin{aligned}
\frac{\partial L_i}{\partial \mu^q_\beta} &= \sum_j \psi^\tau_{ij}\psi^\omega_i (y_{ij} - X_{ij}^\top \mu^q_\beta - \mu^q_{\nu_i}) X_{ij} \\
\frac{\partial L_i}{\partial \mu^q_\alpha} &= \frac{1}{2}[\psi^\alpha_i ({\mu^q_{\nu_i}}^2 + \Sigma^q_{\nu_i}) - 1] U_i \\
\frac{\partial L_i}{\partial \mu^q_\tau} &= \frac{1}{2}\sum_j (\psi^\tau_{ij}\psi^\omega_i h_{ij} - 1) W_{ij}
\end{aligned}
$$

B.2 The Bread Matrix $\hat{A}$

The bread matrix is $\hat{A} = \sum_i \hat{H}_i + \frac{\partial^2}{\partial \theta^2} L_{\text{priors}}$

$$L_{\text{priors}} = \mathbb{E}_q[\log p(\beta) + \log p(\alpha) + \log p(\tau)]$$

Therefore, the Hessian of L_{priors} is:

$$\frac{\partial^2}{\partial \theta^2} L_{\text{priors}} = \text{diag}(\frac{-1}{\sigma^2_\beta}\mathcal{I}, \frac{-1}{\sigma^2_\alpha}\mathcal{I}, \frac{-1}{\sigma^2_\tau}\mathcal{I})$$

And the adjusted Hessian is $\hat{H}_i = \frac{\partial^2 L_i}{\partial \theta^2} - \frac{\partial^2 L_i}{\partial \theta \partial \zeta_i}[\frac{\partial^2 L_i}{\partial \zeta_i^2}]^{-1}\frac{\partial^2 L_i}{\partial \zeta_i \partial \theta}$.

$$\frac{\partial^2 L_i}{\partial \zeta_i^2} = \begin{bmatrix} \frac{\partial^2 L_i}{\partial {\mu^q_{\nu_i}}^2} & \frac{\partial^2 L_i}{\partial \mu^q_{\nu_i} \mu^q_{\omega_i}} \\ \frac{\partial^2 L_i}{\partial \mu^q_{\omega_i} \mu^q_{\nu_i}} & \frac{\partial^2 L_i}{\partial {\mu^q_{\omega_i}}^2} \end{bmatrix}$$

1. $\frac{\partial^2 L_i}{\partial {\mu^q_{\nu_i}}^2} = -\psi^\alpha_i - \sum_j \psi^\tau_{ij}\psi^\omega_i$

2. $\frac{\partial^2 L_i}{\partial {\mu^q_{\omega_i}}^2} = -\frac{A^q_{\sigma^2_\omega}}{B^q_{\sigma^2_\omega}} - \frac{1}{2}\sum_j \psi^\tau_{ij}\psi^\omega_i h_{ij}$

3. $-\sum_j \psi^\tau_{ij}\psi^\omega_i (y_{ij} - X_{ij}^\top \mu^q_\beta - \mu^q_{\nu_i})$

$$\frac{\partial^2 L_i}{\partial \theta^2} = \begin{bmatrix} \frac{\partial^2 L_i}{\partial {\mu^q_\beta}^2} & \frac{\partial^2 L_i}{\partial \mu^q_\beta \mu^q_\alpha} & \frac{\partial^2 L_i}{\partial \mu^q_\beta \mu^q_\tau} \\ \frac{\partial^2 L_i}{\partial \mu^q_\alpha \mu^q_\beta} & \frac{\partial^2 L_i}{\partial {\mu^q_\alpha}^2} & \frac{\partial^2 L_i}{\partial \mu^q_\alpha \mu^q_\tau} \\ \frac{\partial^2 L_i}{\partial \mu^q_\tau \mu^q_\beta} & \frac{\partial^2 L_i}{\partial \mu^q_\tau \mu^q_\alpha} & \frac{\partial^2 L_i}{\partial {\mu^q_\tau}^2} \end{bmatrix}$$

1. $\frac{\partial^2 L_i}{\partial {\mu^q_\beta}^2} = \frac{-1}{\sigma^2_\beta}\mathcal{I} - \sum_{i,j} \psi^\tau_{ij}\psi^\omega_i X_{ij} X_{ij}^\top$

2. $\frac{\partial^2 L_i}{\partial {\mu^q_\alpha}^2} = \frac{-1}{\sigma^2_\alpha}\mathcal{I} - \frac{1}{2}\sum_i \psi^\alpha_i ({\mu^q_{\nu_i}}^2 + \Sigma^q_{\nu_i}) U_i U_i^\top$

3. $\frac{\partial^2 L_i}{\partial {\mu^q_\tau}^2} = \frac{-1}{\sigma^2_\tau}\mathcal{I} - \frac{1}{2}\sum_{i,j} \psi^\tau_{ij}\psi^\omega_i h_{ij} W_{ij} W_{ij}^\top$

4. $\frac{\partial^2 L_i}{\partial \mu^q_\beta \mu^q_\alpha} = 0$

5. $\frac{\partial^2 L_i}{\partial \mu^q_\beta \mu^q_\tau} = -\sum_j \psi^\tau_{ij}\psi^\omega_i (y_{ij} - X_{ij}^\top \mu^q_\beta - \mu^q_{\nu_i}) X_{ij} W_{ij}^\top$

6. $\frac{\partial^2 L_i}{\partial \mu^q_\alpha \mu^q_\tau} = 0$

$$\frac{\partial^2 L_i}{\partial \theta \partial \zeta_i} = \begin{bmatrix} \frac{\partial^2 L_i}{\partial \mu^q_\beta \mu^q_{\nu_i}} & \frac{\partial^2 L_i}{\partial \mu^q_\beta \mu^q_{\omega_i}} \\ \frac{\partial^2 L_i}{\partial \mu^q_\alpha \mu^q_{\nu_i}} & \frac{\partial^2 L_i}{\partial \mu^q_\alpha \mu^q_{\omega_i}} \\ \frac{\partial^2 L_i}{\partial \mu^q_\tau \mu^q_{\nu_i}} & \frac{\partial^2 L_i}{\partial \mu^q_\tau \mu^q_{\omega_i}} \end{bmatrix}$$

1. $\frac{\partial^2 L_i}{\partial \mu^q_\beta \mu^q_{\nu_i}} = -\sum_j \psi^\tau_{ij}\psi^\omega_i X_{ij}$

2. $\frac{\partial^2 L_i}{\partial \mu^q_\beta \mu^q_{\omega_i}} = -\sum_j \psi^\tau_{ij}\psi^\omega_i (y_{ij} - X_{ij}^\top \mu^q_\beta - \mu^q_{\nu_i}) X_{ij}$

3. $\frac{\partial^2 L_i}{\partial \mu^q_\alpha \mu^q_{\nu_i}} = \psi^\alpha_i \mu^q_{\nu_i} U_i$

4. $\frac{\partial^2 L_i}{\partial \mu^q_\alpha \mu^q_{\omega_i}} = 0$

5. $\frac{\partial^2 L_i}{\partial \mu^q_\tau \mu^q_{\nu_i}} = -\sum_j \psi^\tau_{ij}\psi^\omega_i (y_{ij} - X_{ij}^\top \mu^q_\beta - \mu^q_{\nu_i}) W_{ij}$

6. $\frac{\partial^2 L_i}{\partial \mu^q_\tau \mu^q_{\omega_i}} = \frac{-1}{2}\sum_j \psi^\tau_{ij}\psi^\omega_i h_{ij} W_{ij}$

Finally, we combine the meat and bread matrices with

$$\hat{V} = \hat{A}^{-1}\hat{B}\hat{A}^{-1}$$

We can obtain the asymptotic covariance matrix, $\hat{V}$, using this formula. The robust standard errors can then be estimated by taking the square root of each diagonal element of $\hat{V}$.
